# Potential Role of Antioxidant and Anti-Inflammatory Therapies to Prevent Severe SARS-Cov-2 Complications

**DOI:** 10.3390/antiox10020272

**Published:** 2021-02-10

**Authors:** Anna M. Fratta Pasini, Chiara Stranieri, Luciano Cominacini, Chiara Mozzini

**Affiliations:** Section of General Medicine and Atherothrombotic and Degenerative Diseases, Department of Medicine, University of Verona, Policlinico G.B. Rossi, Piazzale L.A. Scuro 10, 37134 Verona, Italy; chiara.stranieri@univr.it (C.S.); luciano.cominacini@univr.it (L.C.); chiara.mozzini@univr.it (C.M.)

**Keywords:** SARS-CoV-2, oxidative stress, inflammation, NRF2, NF-*k*B, adjuvant treatments

## Abstract

The coronavirus disease 2019 (COVID-19) pandemic is caused by a novel severe acute respiratory syndrome (SARS)-like coronavirus (SARS-CoV-2). Here, we review the molecular pathogenesis of SARS-CoV-2 and its relationship with oxidative stress (OS) and inflammation. Furthermore, we analyze the potential role of antioxidant and anti-inflammatory therapies to prevent severe complications. OS has a potential key role in the COVID-19 pathogenesis by triggering the NOD-like receptor family pyrin domain containing 3 inflammasome and nuclear factor-kB (NF-kB). While exposure to many pro-oxidants usually induces nuclear factor erythroid 2 p45-related factor2 (NRF2) activation and upregulation of antioxidant related elements expression, respiratory viral infections often inhibit NRF2 and/or activate NF-*k*B pathways, resulting in inflammation and oxidative injury. Hence, the use of radical scavengers like N-acetylcysteine and vitamin C, as well as of steroids and inflammasome inhibitors, has been proposed. The NRF2 pathway has been shown to be suppressed in severe SARS-CoV-2 patients. Pharmacological NRF2 inducers have been reported to inhibit SARS-CoV-2 replication, the inflammatory response, and transmembrane protease serine 2 activation, which for the entry of SARS-CoV-2 into the host cells through the angiotensin converting enzyme 2 receptor. Thus, NRF2 activation may represent a potential path out of the woods in COVID-19 pandemic.

## 1. Introduction

The coronavirus disease 2019 (COVID-19) pandemic is caused by a novel severe acute respiratory syndrome (SARS)-like coronavirus (SARS-CoV-2) [1].

SARS-CoV-2 is an enveloped, non-segmented, positive sense RNA virus, widely distributed in humans and other mammals [2,3]. SARS-CoV-2 is dissimilar from the coronaviruses recognized to induce the ordinary cold, but it has been shown to have the same characteristics as the zoonotic SARS coronavirus (SARS-CoV) [4] and the Middle East respiratory syndrome (MERS) coronavirus [5]. Patients affected by COVID-19 often display no symptoms or mild symptoms (fever, cough, myalgia, and fatigue) and usually have a good prognosis. Many of these cases, however, progress to a more severe form of the illness, especially in older men experiencing other contemporary serious diseases [2,6,7,8]. Severe patients can suffer from symptoms correlated with lung [2,8,9], heart [8,10,11], kidney [8,12,13], neurological [14,15], gastrointestinal [16] and liver [9,16,17,18] injuries. Furthermore, there may be immune [9,12,19,20] and coagulation [21,22] impairment. Globally, as of December 27, 2020, there have been 79,232,555 confirmed COVID-19 cases, including 1,754,493 deaths [23].

Angiotensin converting enzyme 2 (ACE2) offers an access receptor for SARS-CoV-2 and SARS-CoV in humans by binding to the viral membrane spike (S) protein [24,25]. The quick recognition of ACE2 as SARS-CoV-2 receptor is mostly attributable to its recognition as the receptor for SARS-CoV about 17 years ago. In that case, ACE2 was recognized as the functional receptor for SARS-CoV after the fusion protein gene of SARS-CoV was reported [26]. By means of in vitro studies, Li et al. [27] found that: (1) ACE2 attached to the SARS-CoV S1 protein; (2) a soluble variety of ACE2, but not ACE1, inhibited the binding of the S1 protein with ACE2; (3) SARS-CoV reproduced in a very intense manner in ACE2-transfected, but not mock-transfected, cells. Furthermore, studies in vivo have clearly shown that ACE2 is a pivotal SARS-CoV receptor [28]. Here, we review the molecular pathogenesis of SARS-CoV-2 and its relationship with oxidative stress (OS) and inflammation. Furthermore, we analyze the potential role of antioxidant and anti-inflammatory therapies to prevent severe complications.

## 2. SARS-CoV-2 Cell Entry Mechanisms

### 2.1. SARS-CoV-2 Structural Basis

**Figure 1:**
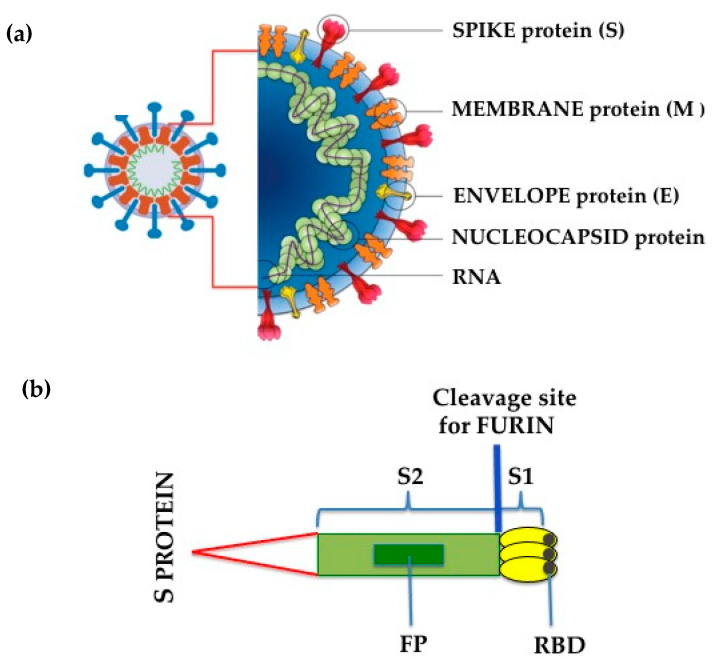
(**a**) SARS-CoV-2 structure; (**b**) Schematic drawing of SARS-CoV-2 Spike (S) protein. S1, receptor-binding subunit; S2, membrane fusion subunit; FP, fusion protein; RBD, receptor binding domain.

Like SARS-CoV, SARS-CoV-2 has four principal structural proteins: spike (S), envelope (E), membrane (M) and nucleocapsid (N), together with several additional proteins [29,30] (Figure 1). The S glycoprotein is a transmembrane protein (molecular weight of about 150 kDa) found in the virus outer portion [31]. Like SARS-CoV, S protein occurs as a trimer, with three receptor-binding S1 heads being placed on top of a membrane fusion S2 stalk [31] (Figure 1). S1, which binds to the peptidase domain of ACE2, is called the receptor-binding domain (RBD), while S2 catalyzes the membrane fusion, thus releasing the genetic material into the cells [31]. The crystal structures of the RBD of the S protein of SARS-CoV-2, both non-complexed [32] (protein data bank code 6VXX, https://www.rcsb.org (accessed on 31 December 2020)) or complexed with human ACE2 [33] (protein data bank code 6M0J, https://www.rcsb.org (accessed on 31 December 2020)) have been published previously. Recent studies, however, have established that there are slight differences between SARS-CoV-2 and SARS-CoV in receptor recognition [34]; these dissimilarities allow SARS-CoV-2 RBD to possess a slightly higher ACE2 receptor affinity than RBD of SARS-CoV [31], even though it results in being less accessible [32,35]. To retain its elevated infectivity despite a low accessibility, SARS-CoV-2 uses activation of host proteases, and this process crucially determines the infectivity and pathogenesis of SARS-CoV-2 infection [31]. In this context, it has previously been established that the pre-activation of furin, a host proprotein convertase [35,36], increases SARS-CoV-2 entrance into cells expressing ACE2 receptor by binding to a polybasic sequence motif at the S1/S2 border of the virus [31]. Furin-cleaved substrates then link to neuropilin-1 (NRP1), facilitating SARS-CoV-2 infectivity [36,37]. Moreover, transmembrane protease serine 2 (TMPRSS2) and lysosomal cathepsins, in addition to forcing SARS-CoV-2 entrance, have an additional impact with furin on SARS-CoV-2 entry [31]. Entered-SARS-CoV-2 will subsequently release its genomic material in the cytoplasm and be translated into the nuclei [38].

### 2.2. Structural Basis and Function of ACE2 Receptor

The renin-angiotensin system (RAS) plays a role in controlling blood volume and systemic vascular resistance, which at the same time affect cardiac output and arterial pressure [39]. ACE, a dipeptidyl carboxypeptidase in the RAS, converts the inactive angiotensin (Ang) I into the active and effective vasoconstrictor Ang II and inhibits the vasodilator Bradykinin [40]. ACE2 counterbalances ACE to a great extent by converting Ang I into Ang 1–9, an inert variety of Ang. It can also break down and hydrolyze the vasoconstrictor Ang II, into Ang 1–7, which acts as a strong vasodilator [41]. The ACE2 crystal structure and RBD of the S protein of SARS-CoV-2 complexed with human ACE2 have previously been reported [33,42] (protein data bank codes 1R42 and 6M0J, respectively, https://www.rcsb.org (accessed on 31 December 2020)). As just reviewed [43,44], ACE2 has multiple crucial protecting roles against hypertension, cardiovascular and lung diseases, and diabetes mellitus. Furthermore, the control of gut dysbiosis and vascular permeability by ACE2 has come out as an intrinsic mechanism of pulmonary hypertension and diabetes-related cardiovascular complications [44].

Very recently, ACE2 has been garnering widespread interest as a functional SARS-CoV-2 and SARS-CoV virus receptor by binding to the viral S protein, in this way contributing to pathogenesis of SARS [11,24,25,27]. ACE2 is ubiquitously expressed, with the highest levels in the epithelial cells of the lung, kidney and cardiomyocytes [45], although there is no lack of discordant voices, mostly for lung tissue [46]. Furthermore, recent studies based on single-cell RNA-sequence (scRNA-seq) data analysis have reported that ACE2 is widespread in many organs, including the lungs, heart, esophagus, kidneys, bladder, ileum, oral mucosa, and, particularly in the case of type II alveolar cells, cardiomyocytes, kidney proximal tubule cells, ileum and esophagus epithelial cells, and bladder urothelial cells [47]. Thank to this diffuse presence, ACE2 is involved in virus infection and diffusion. In addition, it has previously been found that infection with SARS-CoV and SARS-CoV-2 causes ACE2 shedding with subsequent downregulation of surface ACE2 expression [28,48]. In this context, in a small group of severe COVID-19 patients, Ang II plasma concentration was found to be significantly higher than in healthy controls [49], strengthening the hypothesis of a direct link between tissue ACE2 downregulation with systemic RAS imbalance. 

**Figure 2:**
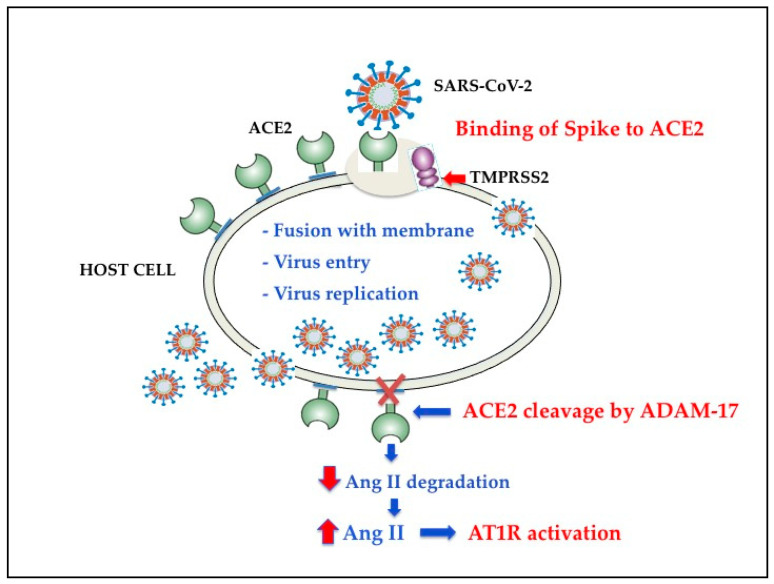
Schematic diagram of SARS-CoV-2 effects on renin angiotensin system. ACE, angiotensin-converting enzyme; ACE2, angiotensin-converting enzyme 2; Ang II, Angiotensin II; Adam-17, a disintegrin and metalloproteinase17; AT1R, angiotensin II type-1 receptor; TMPRSS2, transmembrane protease serine 2.

As shown in Figure 2, recent evidence has shown that ectodomain shedding of ACE2 is mediated by ADAM17 (a disintegrin and metalloproteinase17), which in turn is upregulated by endocytosed SARS-CoV-2 S proteins [50] and other mechanisms [51,52,53,54].

The available body of facts indicates that Ang II binding to AT1R also controls the activation of nicotinamide adenine dinucleotide phosphate (NADPH) oxidases [NOX] [54,55], one of the most important determinants of reactive oxygen species (ROS) generation. Hence, the SARS-CoV-2-induced ACE2 downregulation increases the binding of Ang II to AT1R, which, by triggering NOX, causes oxidative stress (OS) and inflammation in accordance with the COVID-19 severity [46]. 

## 3. TMPRSS2 and SARS-CoV-2

One crucial discovery in learning how SARS-CoV-2 enters into the cells involves the role of TMPRSS2, a cell-surface protein [56] (Figure 2) that was identified in 2001 in the epithelia of the gastrointestinal, urogenital, and respiratory tracts of mouse and humans, although TMPRSS2 expression in human dominates in the prostate [57]. The crystal structure of TMPRSS2 has previously been published ([58], protein data bank code 5AFW, https://www.rcsb.org (accessed on 31 December 2020)). As for the expression of TMPRSS2 in the lung and bronchial branches, a very recent study using the scRNA-seq method showed that the highest expression of TMPRSS2 was in alveolar type 2 cells. Interestingly, these cells also presented the greatest expression of ACE2 [59,60,61,62]. Since SARS-CoV-2 has a furin cleavage site in its S protein, with the potential to increase SARS-CoV-2 binding to ACE2 receptor [31], the authors also detected a preference for co-expression for any association of ACE2, TMPRSS2 and/or furin expression [59]. Accordingly, the priming of SARS-CoV-2 S protein by furin would hypothetically make many more cells susceptible to infection, as compared to S protein priming by TMPRSS2 alone [59]. Furin activity first causes the generation of two non-covalently associated proteins, S1 and S2 [32,60,61], with the TMPRSS2 further priming S2 [57]. Then, the C terminus of the S1 protein may bind to the NRP1, which significantly potentiates SARS-CoV-2 infectivity [36,37]. In this context, however, it has been reported that cells expressing NRP1 alone only play a small part in SARS-CoV-2 infection [36], whereas its co-expression with ACE2 and TMPRSS2 greatly intensified infection [36]. 

Finally, it has to be pointed out that TMPRSS2 has been identified in prostate cancer, and that its expression was upregulated by androgens [62,63]. Previous reports showed that androgen receptors are expressed in the human respiratory tract epithelium, mainly in type 2 alveolar and bronchial epithelial cells [64]. Since growing data support the concept that male gender is a factor associated with a significantly increased risk of severe events and death from COVID-19 [12,65,66], the strong up-regulation of TMPRSS2 by androgens raises the theory that the male prevalence in the COVID-19 pandemic could partially be explained by androgen-driven TMPRSS2 increase [67]. The available data on this point, however, are discrepant [67], and further studies are needed to fully clarify this topic.

## 4. Oxidative Stress and Inflammation Associated with SARS-CoV-2 Infection

### 4.1. Oxidative Stress (OS) in SARS-CoV-2 Infection

**Figure 3:**
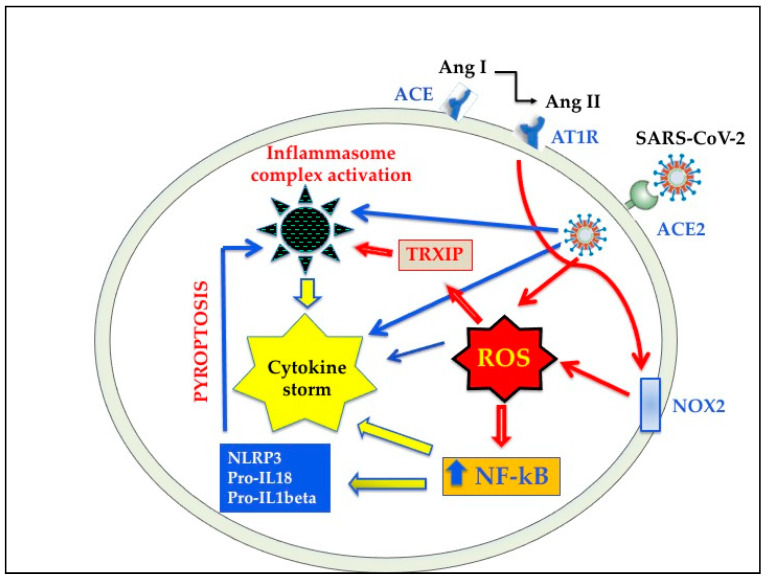
Oxidative stress and inflammation induced by SARS-CoV-2 infection. ACE, angiotensin-converting enzyme; ACE2, angiotensin-converting enzyme 2; AT1R, angiotensin II type-1 receptor; NOX2, NADPH oxidase 2, NF-kB, nuclear factor kB; ROS, reactive oxygen species; TRXIP, thioredoxin interacting/inhibiting protein; NLRP3, NOD-like receptor protein 3.

It is known that OS arises whenever there is an imbalance between ROS formation and antioxidant defenses. Alterations of the redox state towards oxidant conditions in infected cells is one of the key events in respiratory viral infections that is linked to inflammation and subsequent tissue damage [68,69,70]. Recent evidence indicates that OS play a crucial role also in COVID-19 infection [71,72,73,74,75]. Several in vitro and in vivo studies have shown that ROS overproduction induced by respiratory viruses is partially mediated by the activity of NOX (reviewed in [69]). As reported above, ACE2 shedding caused by SARS-CoV-2 fusion may be strictly related to RAS imbalance [43,47], and there is now evidence that Ang II controls NOX activation [54,55] (Figure 3). It has been suggested that NOX2 is a key event in killing bacteria and fungi, but it does not efficiently function against viruses [71]. In this regard, a recent study shows that OS induced by NOX2 activation is linked with severe clinical outcome and thrombotic events in COVID-19 patients [76]. ACE2 downregulation and OS are also associated with endothelial dysfunction via NOX activation and reduced availability of nitric oxide [77]. Furthermore, oxidized phospholipids (OxPLs), which are a product of OS and have been detected in the lungs of SARS-CoV patients [78], were found to be one of the main triggers of acute lung injury. As a matter of fact, OxPLs were shown to promote tissue factor expression [78], to activate endothelial cells to recruit monocytes [79,80], and to trigger macrophage activation through Nuclear Factor-κB (NF-kB) pathway [78]. It remains to be elucidated whether analogous pathways are also involved in SARS CoV-2 infection. 

It is well recognized that the levels of cellular free iron must be tightly regulated to avoid ROS generation via the Fenton reaction [81]. Upon SARS-CoV-2 infection, IL-6 in the cytokine storm increases ferritin and the production of hepcidin, which plays a main role in iron regulation. Since iron is sequestered by hepcidin in the enterocytes and macrophages, intracellular ferritin is augmented, leading to a reduced iron efflux from the cells. The stored iron may increase intracellular labile iron (II) pool and Fenton reaction, producing lipid ROS, and lead to ferroptosis, a novel form of regulated cell death [81]. In COVID-19 patients, the documented iron metabolism alterations may cause iron accumulation and overload, triggering ferroptosis in the cells of multiple organs [82,83]. 

Many lines of evidence show that viruses may also generate OS per se [69,70]. With regard to SARS-CoV, the viral protease 3CLpro has been previously shown to increase ROS generation in HL-CZ cells, with subsequent cell apoptosis and NF-kB-activation [84]. Another SARS-CoV protease, the 3a protein, has been linked with mitochondrial cell death pathway activation by triggering OS [69]. 

The mitochondrial respiratory chain is the main and most significant source of cellular ROS. However, while mitochondrial ROS production was once seen as merely an accidental by-product of oxygen metabolism of mitochondrial respiratory chain, it is now clear that ROS contribute to various signaling pathways [85]. Depending on the context and triggering stimuli, mitochondrial ROS production can lead to different cellular responses such as adaptation to hypoxia, differentiation, autophagy, inflammation, or to an immune response [86]. In general, viruses can modify mitochondrial dynamics in a highly specific manner so that they can successfully replicate [87]. Among the different mechanisms implicated, there are mitochondrial DNA damage, changes in mitochondrial membrane potential, variations in mitochondrial metabolic pathways and calcium homeostasis, modifications in number and distribution of mitochondria into the cells, weakening of antioxidant defense, and augmented OS [87,88]. Upon infection, viruses completely rely on host cell molecular machinery to survive and replicate [87,88]. Mitochondria defend host cells from SARS-CoV-2 virus through several mechanisms including cellular apoptosis, ROS production, autophagy, mitochondrial antiviral signaling system (MAVS) activation, DNA-dependent immune activation, and other things [89]. Current knowledge of how SARS-CoV-2 infection affects mitochondria and their ROS generation is limited. A prior study on SARS-CoV [90] showed that open reading frame-9b (Orf9b), one of the accessory proteins of the virus [91], alters host cell mitochondria morphology, disrupts MAVS, inhibits interferon (IFN) production and enhances autophagy, a cellular mechanism activated by ROS [92]. Consistent with the findings of SARS-CoV, Gordon et al. [93] recently reported that SARS-CoV-2 Orf9b interacts with mitochondrial translocase of outer membrane (TOM)70, although the functional consequences of this association were not examined. Very recently, Jiang et al. [94] reported that SARS-CoV-2 Orf9b localizes to the membrane of mitochondria and suppresses IFN-I response through association with TOM70. The altered activity of TOM70, by reducing constitutive calcium transfer to mitochondria, dampens mitochondrial respiration, affects cell bioenergetics, and induces autophagy [95].

During viral infections beyond an over-production of ROS, there is a decreased antioxidant defense, mainly Glutathione (GSH) depletion, in the host cells that directly or indirectly favor viral replication [96]. GSH, a tripeptide consisting of cysteine, glycine, and glutamate, is the main intracellular antioxidant that applies an efficient buffering role against ROS, through the thiol group of its cysteine which oxidizes to the disulfide form, then reduced back to the thiol form by glutathione reductase [70]. 

It has a principal role in cellular signaling and processes, as well as innate immune response to viruses [70].

A significant elevation in blood serum GSH reductase, derived from OS imbalance, was found in COVID-19 patients, especially when admitted to the intensive care unit [97]. Additionally, mounting evidence supports the concept that the reduced levels of GSH may underlie the COVID-19 severe clinical outcome and death [98].

### 4.2. Cross Talks between Oxidative Stress and Inflammation in SARS-CoV-2 Infection

Several studies have demonstrated that SARS-CoV-2 infection and the destruction of lung cells causes a local immune response, recruiting macrophages and monocytes that reply to the infection, release cytokines and prime adaptive T and B cell immune responses. In most patients, this process overcomes the infection. However, sometimes, a dysfunctional immune response occurs, which leads to a cytokine storm that mediates general lung inflammation [2,99,100]. Increased plasma concentrations of inflammatory markers such as C-reactive protein and ferritin, of many cytokines such as TNF-alpha, IL-1beta, IL-6 and IL-8, and chemokines such as MCP1, together with increased neutrophils/lymphocytes ratio [11,99,100], have been associated with gravity of SARS-CoV-2 infection and death [2,19,101]. 

SARS-CoV-2 infection in type 2 alveolar and other cells activates NOD-like receptor protein 3 (NLRP3), an element of the innate immune system that acts as a pattern recognition receptor that recognizes damage-associated molecular patterns (DAMPs) and pathogen-associated molecular patterns (PAMPs) [102] and takes part in multiprotein complexes called inflammasomes, which bring together sensor proteins (like NLRP3) [103,104]. NLRP3 inflammasome is very often associated with cellular death by apoptosis and pyroptosis [105,106,107], an inflammatory form of programmed cell death [108] that releases large amounts of pro-inflammatory mediators [109]. Accumulating data have established a causal role between the pyroptosis of alveolar type 2, endothelial and immune cells and the progression of lung damage [110,111,112,113,114,115]. The contemporary activation of alveolar macrophages further produces large amounts of proinflammatory cytokines and chemokines [116,117], which activate endothelial cells [118,119], platelets [120,121] and neutrophils [122,123] generating platelet neutrophil complexes at endothelium surface [124,125]. This sequestration of platelet neutrophil complexes in the pulmonary vasculature is the prelude of a highly inflammatory and pro-coagulant situation, a state called immunothrombosis [119,126,127]. Convincing evidence shows that immunothrombosis is a pivotal determinant of micro-thrombi and micro-emboli generation in the alveolar capillary circulation [128,129], of fibrin deposition within the alveoli, and in some cases of disseminated intravascular coagulation [130,131,132]. Furthermore, the huge associated increase of activated neutrophils in lung interstitial tissue and alveoli [133] can discharge high levels of extremely cytotoxic neutrophil extracellular traps [133]. These events play a crucial part in determining intra-lung cytokine storm and the consequent tissue damage that is a peculiarity of ARDS, an inflammatory disease with pulmonary epithelial and capillary endothelial cells dysfunction, alveolar macrophages and neutrophils infiltration, cell death, and fibrosis [134].

While it is likely that lung and other tissue damages in SARS-CoV-2 infection are the results of multifactorial mechanisms, very recent studies indicate that ROS may play a major role in the initiation and progression of this inflammatory process [135,136]. In this context, it has been reported that OS triggers the NLRP3 inflammasome [137,138]. Although it is conceivable that other pathological pathways participate in NLRP3 induction [139,140,141], OS activates NLRP3 inflammasome through NF-*k*B and thioredoxin interacting/inhibiting protein [135,136,137,138,142,143] activation. In addition, NF-kB up-regulates IL-18 and IL-1beta expression, further increasing NLRP3 inflammasome [141,143,144], as shown in Figure 3. This OS-induced overactivation of NLRP3 inflammasome may play a key role in the pathogenesis of severe SARS-CoV-2 infection. In fact, when the innate response cannot clear the infection, the resulting NLRP3 hyperactivation is harmful, leading to perturbation of mitochondrial function, the release of DAMPS and mounting pyroptosis [102,103,104] determining virus propagation and massive destruction of affected tissues [145,146].

## 5. Rationale for Antioxidant and Anti-Inflammatory Therapies against COVID-19 Complications

### 5.1. Radical Scavengers

Modulation of the intracellular redox state is a pivotal strategy that viruses use to manipulate host cell machinery to their advantage [68]. Accordingly, recent studies have focused on redox-sensitive pathways as novel cell-based targets for therapies designed to stop both viral replication and virus-induced inflammation. Since respiratory viruses not only improve ROS production but also impair cellular defense systems, the use of radical scavengers has long been considered to be a potential therapeutical approach [69,70]. 

In the COVID-19 pandemic, the search for alternative therapies for the treatment of coronavirus diseases is of great importance; in this context, antioxidant therapies have been proposed as a potential treatment, preventive and/or adjuvant against SARS-CoV-2.

The most encouraging compounds comprise GSH and its precursor N-acetylcysteine (NAC). NAC is a natural antioxidant derived from plants especially from the Allium species, whose thiol group directly scavenges ROS and helps GSH synthesis [147]. Since NAC is applied in a broad range of conditions to restore GSH depletion it has been suggested as a nutraceutical that might aid the control of RNA viruses including influenza and coronavirus [148]. 

It is well recognized that the interaction of viral S protein with ACE2 is an important step in the viral replication cycle [24,25]. The RBD of the viral S protein and ACE2 have several cysteine residues [149,150]; interestingly, it has recently been found that the binding affinity is significantly impaired when all the disulfide bonds of both ACE2 and SARS-CoV/CoV-2 S proteins are reduced to thiol groups [149,150]. These facts are consistent with the view that the reduction of disulfides into sulfhydryl groups completely impairs the binding of SARS-CoV/CoV-2 S protein to ACE2 and provide a molecular basis for the COVID-19 infection severity due to OS [149,150].

Based on the protective role of NAC in experimental models of influenza and other viruses [151,152], it has recently been suggested that NAC may be used both in the COVID-19 prevention and in therapy [153]. Recently, NAC has been demonstrated to also exert protective mechanisms against a variety of COVID-19 associated conditions including cardiovascular diseases [154]. Administration of NAC has also been considered among the possible strategies aimed at protecting endothelial function and restricting microthrombosis in severe forms of the COVID-19 disease [155]. A potential role of NAC and copper in combination with candidate antiviral treatments against SARS-CoV-2, such as remdesivir, has been hypothesized based on a systematic literature search [156]. Clearly, these possible anti-COVID-19 mechanisms and properties of NAC need to be confirmed in controlled clinical trials [157,158]. In particular, the results of “A Study of *N*-acetylcysteine in Patients with COVID-19 Infection” (NCT04374461) aimed at evaluating the effect of NAC (iv; 6 g/day) administration as an adjuvant treatment in patients with severe COVID-19 symptoms will help to corroborate the potential therapeutic properties of this thiol in COVID 19 patients. The patients were enrolled into two separate arms and the mechanically ventilated and/or managed in a critical-care arm is closed to accrual as of September 2020 [159].

A further mechanism that has recently been proposed is the possibility that NAC further improves the stimulation of Nuclear factor erythroid 2 p45-related factor2 (NRF2) by OS, which promotes the transcription of phase II enzyme genes and downregulates inflammation [160]. At the same time, NAC prevents the OS-mediated activation of NF-κB and biochemical pathways upregulating pro-inflammatory genes [161]. NAC also reduced the intracellular hydrogen peroxide concentration and restored the intracellular total thiol contents by impeding NF-κB translocation to the cellular nucleus and phosphorylation of p38 mitogen-activated protein kinase [162].

Taken together, the results of experimental and clinical studies available so far indicate that NAC acts in a variety of potential therapeutic target pathways involved in the pathophysiology of SARS-CoV-2 infection. 

It is well recognized that during viral infections, intracellular GSH depletion is a common event that is central for viral replication [97], and several in vitro and in vivo studies have found that GSH administration blocks viral replication through redox state modulation [70]. An improving GSH molecule is I-152, a combination of NAC and s-acetyl-mercaptoethylamine (cysteamine, MEA) that can release NAC and MEA thus increasing GSH. Its antiviral efficacy has been evidenced in in vitro and in vivo models [163]. Interestingly, a case report study showed that the repetitive use of both 2000 mg of oral administration and intravenous injection of GSH was effective at relieving COVID-19 severe respiratory symptoms, demonstrating for the first time the usefulness of this antioxidant therapy for COVID-19 patients [164]. 

As far as Vitamin C is concerned, its important anti-inflammatory, immunomodulating, antioxidant, antithrombotic and antiviral properties are well known [165] as a contributor in cytokine down-regulation and ROS lowering via attenuation of NF-kB activation.

Vitamin C deficiency in gulonolactone Loxidase-knockout mice [166] showed enhanced Neutrophil Extracellular Traps (NETs) in the lungs of septic animals and increased circulating cell-free DNA suggesting that vitamin C is a novel regulator of NETosis, which is a particular cell death [167] implicated in the response to fighting COVID-19 [168].

The pharmacological effects of Vitamin C that could make it a potential option for prevention and treatment of COVID-19 have recently been reviewed [169]. Clinicians using intravenous Vitamin C in severely ill COVID-19 patients have reported positive clinical effects upon administration of 3 g every 6 h, together with steroids and anti-coagulants [170]. There are currently several clinical trials registered on Clinicaltrials.gov investigating Vitamin C (oral or intravenous) with or without other treatments for COVID-19. The largest registered trial is the Lessening Organ Dysfunction with Vitamin C-COVID (LOVIT-COVID) trial in Canada, which is recruiting 800 patients who are randomly assigned to Vitamin C (intravenous, 50 mg/kg every 6 h) or a placebo for 96 h, i.e., equivalent to 15 g/day (NCT04401150). This protocol has also been added as a Vitamin C arm in the Randomized, Embedded, Multifactorial Adaptive Platform Trial for Community-Acquired Pneumonia (REMAP-CAP; NCT02735707). The study design provides further rationale for the use of Vitamin C in COVID-19 patients [171].

A case series of 17 COVID-19 patients who were given 1 g of intravenous Vitamin C every 8 h for 3 days reported decreased mortality, decreased intubation and mechanical ventilation need and a significant decrease in inflammatory markers, including ferritin and D-dimer, and a trend towards decreasing FiO2 requirements [172]. These parameters are under investigation in the “Intravenous Vitamin C Administration in Coronavirus (COVID-19) and Decreased Oxygenation (AVoCaDO), NCT04357782” clinical trial in which subjects administered with intravenous Vitamin C are supposed to be at lower risk of respiratory failure worsening and reduced inflammation markers increase. As of October 13, 2020, recruitment has been completed.

Whether or not Vitamin C supplementation will consistently prevent conversion to the critical phase of COVID-19 has yet to be determined, but given its favorable safety profile and low cost, and the frequency of its deficiency in respiratory infections, it may be worthwhile testing patients’ vitamin C status and treating them.

### 5.2. NRF2 Activators

NRF2 is a leading transcription factor that targets genes coding for antioxidant proteins and detoxification enzymes [161]. NRF2 regulates the basal and induced expression of an array of antioxidant response element (ARE-dependent genes, such as heme-oxygenase (HO)-1) to regulate the physiological and pathophysiological outcomes of oxidant exposure. Under basal conditions, NRF2-dependent transcription is blocked by its negative regulator, Kelch-like enoyl-CoA hydratase-associated protein1 (Keap-1); when cells are exposed to OS or electrophiles, NRF2 accumulates in the nucleus and drives its target genes expression [160].

Several studies support the concept that viral infections interfere with antioxidative systems, causing an imbalance between oxidative and antioxidative status and subsequent oxidative cell injuries [68,69,70,71]. In particular, while exposure to many pro-oxidants induces NRF2 activation and upregulation of ARE gene expression, respiratory viral infections often inhibit NRF2 pathway and/or activate NF-kB transcription factor, resulting in inflammation and oxidative injury [173]. The activation or inhibition of NRF2 in host cells is dependent on factors such as the stage of infection [174] or the peculiar viral propagation mechanisms by which cell death and release of viruses are caused [175]. A first key demonstration that SARS-CoV-2 virus deprives the host cells of this essential cytoprotective pathway stems from the recent evidence indicating that NRF2 pathway was repressed in lung biopsies of patients affected by SARS-CoV-2 infection [176]. 

There is a reciprocal crosstalk between NRF2 and NF-κB when innate immune cells are enrolled in inflamed tissues [177,178,179]. In in vitro studies, subsequent to infection with SARS-CoV, NF-κB was reported to switch on in mice lungs and in human macrophages; on the contrary, NF-κB inhibition decreased inflammation and ameliorates survival in SARS-CoV-infected mice [180,181]. Therefore, while NRF2 suppression may be associated with high-grade NF-kB activation and consequently with inflammation, activation of NRF2 by specific drugs may delimit NF-κB activity in patients with SARS-CoV-2 infection.

Increasing evidence supports the concept that pharmacological activation of NRF2 may be a promising adjuvant therapy against SARS-CoV-2 infection [182]. In particular, NRF2 inducers may protect against the excessive inflammatory response in COVID-19 patients through different mechanisms: host cell protection, anti-inflammatory phenotype activation, thus preventing uncontrolled proinflammatory cytokines production, pyroptosis and viral propagation inhibition [182].

NRF2 can be triggered by pharmacological inducers that target Keap1; in fact, a lot of NRF2 inducers, including dimethyl fumarate (DMF), sulforaphane, and bardoxolone methyl, are electrophiles that alter cysteine sensors of Keap1 and disarm its repressor function [182]. 

An important issue is whether NRF2 activators may reduce SARS-CoV-2 replication. In this context, the NRF2 agonists 4-octyl-itaconate (4-OI) and the clinically approved DMF suppress SARS-CoV-2 replication and the expression of associated inflammatory genes in cultured cells [176]. In the opinion of the authors [176], the fact that 4-OI suppressed to a great degree the IFN antiviral response but maintained the capacity to inhibit viral replication and attenuate the inflammatory response suggests the existence of unrecognized cellular pathways that work independently of IFNs. 

Many reports have described numerous antiviral effects for HO-1 against a broad spectrum of viruses. In many cases, the mechanism of action of HO-1 products has been recognized, showing direct effects on virus components or cellular processes that interfere with virus replication [183]. Although there are no data so far for targeting HO-1 on SARS-CoV-2, it has been proposed that inducing HO-1 expression may avoid SARS-CoV-2-induced lung complications by means of its antiviral, anti-inflammatory, antithrombotic and antifibrotic properties [184].

**Figure 4:**
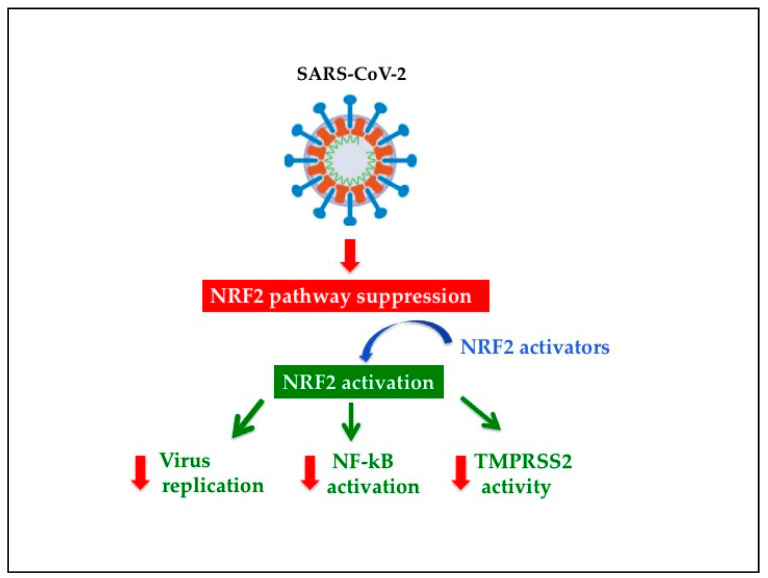
Potential beneficial effects of Nrf2 activators against SARS-CoV2-infection. NRF2, nuclear factor erythroid 2 p45-related factor 2; NF-kB, nuclear Factor kB; TMPRSS2, transmembrane protease serine 2.

Another important point is whether NRF2 can suppress SARS-CoV-2 access into the host cells, and in this scenario, a key role is carried out by TMPRSS2 [56]. PB125, a strong NRF2 inducer, was able to significantly downregulate ACE2 and TMPRSS2 expression in HEPG2 cells [185]. Intriguingly, it also induced a strong upregulation of the human antiprotease plasminogen activator inhibitor-1 (PAI-1) expression, a potent TMPRSS2 inhibitor [186]. Accordingly, the authors suggest that PB125 treatment might reduce the SARS-CoV-2 capacity to bind to a host cell and to provoke S protein activation [185]. In the same study, PB125 was also shown to markedly downregulate genes encoding cytokines [185], many of which were exactly recognized in the cytokine storm seen in lethal cases of COVID-19 [187]. Moreover, it was previously reported that bromhexine, an FDA-approved ingredient in mucolytic cough suppressants, had the capacity to inhibit TMPRSS2 activity and to reduce prostate cancer enlargement and metastases [188]. At present, the mechanism involved in bromhexine-induced TMPRSS2 activity suppression is unknown. However, ambroxol, a metabolite of bromhexine, which has been approved by the FDA and has been established for decades for the treatment of acute and chronic respiratory diseases [189], has also been found to exert an excellent anti-inflammatory and antioxidant activity and to elicit a remarkable induction of NRF2 associated with a concomitant decrease in NF-kB expression in mice [190]. The bromhexine effectiveness in SARS-CoV-2 infection in a small open-label randomized clinical was recently reported by Ansarin et al. [191]. They found that bromhexine administration was associated with a significant reduction in intensive care unit admissions, intubation and death suggesting that TMPRSS2 suppression may contribute to clinically ameliorate SARS-CoV-2 infection (Figure 4).

Finally, DMF, which is now used as an anti-inflammatory drug in relapsing-remitting Multiple Sclerosis [192], could easily be repurposed and verified in clinical trials as a small molecule inhibitor of SARS-CoV-2 replication and inflammation-induced pathology in COVID19 patients. 

Likewise, the wealth of safety and efficacy information for other NRF2 activators, such as sulforaphane and bardoxolone methyl, which are now in advanced clinical trials for other indications, offers a clear means for their testing in COVID-19 randomized clinical trials. If confirmed, this therapeutic strategy could be rapidly mobilized to improve recovery and decrease the need for mechanical ventilation in severe COVID-19 patients, helping to relieve the big strain that is currently being experienced by intensive care units worldwide [182].

### 5.3. Delivery of Soluble ACE2

SARS-CoV-2 infection causes ACE2 shedding from tissue, thus effectively lowering the ACE2 receptor level in infected cells [28,48]. In this regard, it has been suggested that delivery of recombinant ACE2 protein may be a treatment to stop SARS-CoV-2 spreading, and also to preserve RAS system and inhibit ROS generation by NOX [28]. Interestingly, in an in vitro and in vivo study, NOX4-derived ROS production was demonstrated to be modulated by ACE2 [193].

A new in vitro study demonstrated that the fusion protein of recombinant human [rh] ACE2 with a Fc fragment showed high affinity binding to the RBD of SARS-CoV-2 and potently neutralized SARS-CoV-2 entry [194]. In addition, a recent paper strongly supported the efficacy of rhACE2 against SARS-CoV-2 infection [195]. In particular, the authors reported that clinical-grade rh soluble ACE2 exhibited strong inhibitory activity against SARS-CoV-2 in cell cultures and in human blood vessels and kidney engineered copies [184]. Very interestingly, in a recent case report, Zoufaly et al. [196] found that the delivery of rhACE2 in a SARS-CoV-2 patient caused a marked clinical improvement associated with reduction of inflammatory markers and of Ang II with a striking rise of Ang 1–7 and Ang 1–9. Intriguingly, SARS-CoV-2 viremia was significantly reduced after the first day of administration and thereafter it remained undetectable [196]. 

### 5.4. Inhibitors of NLRP3 Inflammasome

Given the strong inflammatory potential of NLRP3 inflammasome in the pathogenesis of different inflammatory diseases, many efforts have been made in the last few years in the search of NLRP3 inhibitors. As recently reviewed [197], many natural products and pharmaceutical drugs have been identified as NLRP3 inhibitors. Among natural and pharmaceutical products, oridonin (derived from Rabdosia rubescens plant) and parthenolide (derived from feverfew plant) as well as Bay 11-7082 have been reported to strongly suppress NLRP3 inflammasome in experimental models [198,199]. Besides inhibiting NLRP3, parthenolide and Bay 11-7082 have also been shown to lower NF-kB activation and to prevent lung inflammation in animals affected by SARS-CoV [199]. Another drug reducing NLRP3 inflammasome activity and IL-1beta secretion in cells infected with RNA viruses is glyburide, a sulfonylurea extensively used in the treatment of type 2 diabetes [200,201]. Likewise, tranilast, a drug used for allergic conditions, was shown to reduce NF-kB activation and NLRP3 assembly in animal models of inflammatory diseases [202]. Similarly, colchicine, a drug used in autoinflammatory diseases for its effect of preventing adhesion and recruitment of neutrophils at endothelial surface [203], can also suppress NLRP3 inflammasome and production of IL-1beta and IL-18 [204]. Finally, mefenamic acid and flufenamic acid, belonging to the group of non-steroidal anti-inflammatory drugs, by inhibiting NLRP3 inflammasome and IL-1beta secretion, have been reported to strongly suppress viral replication independent of their cyclooxygenase-1 mediated anti-inflammatory activity [205,206]. Because of the key role of NLRP3 inflammasome activation in the pathogenesis of SARS-CoVs diseases and the promising results obtained by inhibitors of the NLRP3 inflammasome in pre-clinical and/or clinical studies [197], it can be hypothesized that its inhibition may potentially decrease tissue inflammation also in COVID-19. 

### 5.5. Glucocorticoids (GCs) and Non-Steroidal Anti-Inflammatory Drugs (NSAIDs)

It is well established that GCs control inflammation through pleiotropic mechanisms [207,208]. In particular, GCs block the activation of transcription factors that mediate inflammatory responses, such as NF-kB and activator protein 1 [207], thus suppressing the synthesis of many pro-inflammatory cytokines and of inducible nitric oxide synthase. [207,209]. Furthermore, GCs reduce cyclooxygenase 2 activity by inducing the NF-kB inhibitor glucocorticoid-induced leucine zipper protein, thus weakening prostaglandin release [210]. GCs also inhibit adhesion molecule expression in endothelia cells and integrins in immune cells so diminishing leukocyte recruitment [207,211,212]. In addition, GCs reverse macrophages to an anti-inflammatory state, promote resolution of inflammation, and weaken antigen presentation in dendritic cells [207,213,214,215]. In view of this formidable strength, GCs are considered the cornerstone of the anti-inflammatory and immunosuppressive therapy. At the beginning of COVID-19 pandemic there were many perplexities for handling an infectious disease with potent immunosuppressive agents like GCs. Then, on the basis of the promising results derived from case reports and small observational studies, a series of large-scale randomized clinical trials were started. In the Randomised Evaluation of COVID-19 Therapy (RECOVERY, NCT04381936) trial [216], patients (n. 2104) were randomly assigned to receive oral or intravenous dexamethasone (6 mg once daily) for up to 10 days or to receive usual care (n. 4321). The preliminary results showed that in COVID-19 hospitalized patients, the use of dexamethasone significantly reduced 28-day mortality among those who were receiving either invasive mechanical ventilation or oxygen alone. On the contrary, dexamethasone had no effect among patients not requiring respiratory support. In the prospective meta-analysis of the Rapid Evidence Appraisal for COVID-19 Therapies (REACT) Working Group of the World Health Organization (WHO) [217], the authors analyzed pooled data from seven randomized clinical trials evaluating the efficacy of GCs in 1703 critically ill patients with COVID-19. Similarly to the RECOVERY study [216], the REACT study demonstrated that low-dose dexamethasone, compared with usual care or placebo, reduced all-cause mortality in hospitalized patients with COVID-19 who required respiratory support. Following these studies, the WHO released two recommendations establishing that GCs (dexamethasone per os or intravenously and hydrocortisone intravenously) should be given for 7 to 10 days only in critical and severe COVID-19 patients. 

As for NSAIDs, it has been established that they operate by suppressing cyclooxygenase (COX) 1 and 2, thus limiting the synthesis of prostaglandins, which play a crucial role in the pathogenesis of fever and inflammation [218]. NSAIDs are habitually employed in SARS-CoV-2 infection to lower fever and alleviate muscle pain, but whether NSAIDs are helpful or damaging to COVID-19 patients is currently uncertain, and a cautious strategy is suggested [219,220,221]. Available data so far on the effects of chronic treatment with NSAIDs on SARS-CoV-2 infection are few, and have not been derived from randomized clinical trials. In particular, a large case control study showed that chronic treatment with NSAIDs was not associated with risk of COVID-19 infection or COVID-19 severity [222]. Similar results stem from a recent multicenter retrospective cohort study of hospitalized patients with COVID-19 demonstrating a lack of association between the pre-hospital use of NSAIDs and mortality [223]. Additionally, in a particular setting of COVID-19 patients with chronic inflammatory rheumatic disease, the prior treatment with NSAIDs did not influence the risk of hospitalization [224]. Concerning the potential role of NSAIDs as adjuvant therapy in COVID-19 patients, a recent pilot study showed that adjuvant treatment with celecoxib, a selective inhibitor of COX2, improved the recovery in non-severe and severe cases of SARS-CoV-2 patients and impeded the evolution to a critical step [225].

Although the WHO, the European Medicines Agency (EMA) and the United Kingdom National Health Service have stated that there is currently no scientific evidence that NSAIDs augment the risk or worsen SARS-CoV-2 infection, and that there is no reason for patients who are taking NSAIDs for chronic diseases to stop taking them, from a clinical point of view, it is now recommended that patients with COVID-19 should use paracetamol rather than NSAIDs [226]. This advice is further strengthened by previous clinical trials in non-SARS-CoV-2 pulmonary infectious diseases that have suggested avoiding these drugs (reviewed in [227]). 

## 6. Conclusions

The redox-regulated intracellular pathways triggered and used by viruses may constitute a new and encouraging target for novel approaches in the control and therapy of viral infections. In this context, it has been demonstrated that respiratory viral infections and in particular SARS-CoV-2, despite a dysregulation of ROS production, inhibit NRF2 and activate NF-kB pathways, resulting in inflammation and oxidative injury. The outstanding results from experimental studies available so far clearly indicate the need to also test NRF2 activators in randomized clinical trials in patients with SARS-CoV-2 infection. 

## Abbreviations

ACE	Angiotensin Converting Enzyme
Ang	Angiotensin
AT1R	Angiotensin II Type-1 Receptor
ADAM17	A Disintegrin And Metalloproteinase17
COX	cyclooxygenase
COVID-19	Coronavirus disease 2019
DAMPs	damage-associated molecular patterns
DMF	Dimethyl Fumarate
EMA	European Medicines Agency
FiO2	Fraction Of Inspired Oxygen
FP	Fusion protein
GCs	Glucocorticoids
GSH	Glutathione
HO	Heme-Oxygenase
Hb	Hemoglobin
Keap-1	Kelch-like enoyl-CoA hydratase-associated protein 1
IFN	Interferon
IL	Interleukin
MAVS	Mitochondrial antiviral signaling system;
MCP1	monocyte chemoattractant protein-1
MEA	s-acetyl-mercaptoethylamine
MERS	Middle East respiratory syndrome
NAC	N-acetylcysteine
NETs	Neutrophil extracellular traps
NLRP3	NOD-like receptors protein 3
NRP1	Neuropilin-1
NOX	Nicotinamide adenine dinucleotide phosphate (NADPH) oxidases
NF-kB	Nuclear Factor-κB
NRF2	Nuclear factor erythroid 2 p45-related factor2
NSAIDs	non-steroidal anti-inflammatory drugs
4-OI	4-Octyl-Itaconate
Orf9b	Open reading frame-9b
OS	Oxidative stress
OxPLs	Oxidized phospholipids
PAI-1	Plasminogen Activator Inhibitor-1
PAMPs	Pathogen-associated molecular patterns
RAS	Renin-angiotensin system
RBD	Receptor-binding domain
rh	Recombinant Human
ROS	Reactive oxygen species
SARS-CoV-2	Severe Acute Respiratory Syndrome (SARS)-like Coronavirus
S protein	Viral Membrane Spike protein
S1	Receptor-binding subunit
S2	Membrane fusion subunit
scRNA-seq	single cell RNA-sequence
TRXIP	Thioredoxin Interacting/Inhibiting Protein;
TOM	Translocase of Outer Membrane
TMPRSS2	Transmembrane protease serine 2
WHO	World Health Organization
